# UAV-Based Digital Terrain Model Generation under Leaf-Off Conditions to Support Teak Plantations Inventories in Tropical Dry Forests. A Case of the Coastal Region of Ecuador

**DOI:** 10.3390/s19081934

**Published:** 2019-04-25

**Authors:** Fernando J. Aguilar, José R. Rivas, Abderrahim Nemmaoui, Alberto Peñalver, Manuel A. Aguilar

**Affiliations:** 1 Department of Engineering, University of Almería, Ctra. de Sacramento s/n, La Cañada de San Urbano, 04120 Almería, Spain; an932@ual.es (A.N.); maguilar@ual.es (M.A.A.); 2 Faculty of Technical Education for Development, Santiago de Guayaquil Catholic University, Av. Carlos Julio Arosemena, Guayaquil 090615, Ecuador; jose.rivas02@cu.ucsg.edu.ec (J.R.R.); alberto.penalver01@cu.ucsg.edu.ec (A.P.)

**Keywords:** tropical dry forest, teak plantations, digital terrain model, remote sensing, structure from motion, UAV, forest inventory

## Abstract

Remote sensing is revolutionizing the way in which forests studies are conducted, and recent technological advances, such as Structure from Motion (SfM) photogrammetry from Unmanned Aerial Vehicle (UAV), are providing more efficient methods to assist in REDD (Reducing Emissions from Deforestation and forest Degradation) monitoring and forest sustainable management. The aim of this work was to develop and test a methodology based on SfM from UAV to generate high quality Digital Terrain Models (DTMs) on teak plantations (*Tectona grandis* Linn. F.) situated in the Coastal Region of Ecuador (dry tropical forest). UAV overlapping images were collected using a DJI Phantom 4 Advanced^©^ quadcopter during the dry season (leaf-off phenological stage) over 58 teak square plots of 36 m side belonging to three different plantations located in the province of Guayas (Ecuador). A workflow consisting of SfM absolute image alignment based on field surveyed ground control points, very dense point cloud generation, ground points filtering and outlier removal, and DTM interpolation from labeled ground points, was accomplished. A very accurate Terrestrial Laser Scanning (TLS) derived ground points were employed as ground reference to estimate the UAV-SfM DTM vertical error in each reference plot. The plot-level obtained DTMs presented low vertical bias and random error (−3.1 cm and 11.9 cm on average, respectively), showing statistically significant greater error in those reference plots with basal area and estimated vegetation coverage above 15 m^2^/ha and 60%, respectively. To the best of the authors’ knowledge, this is the first study aimed at monitoring of teak plantations located in dry tropical forests from UAV images. It provides valuable information that recommends carrying out the UAV image capture during the leaf-off season to obtain UAV-SfM derived DTMs suitable to serve as ground reference in supporting teak plantations inventories.

## 1. Introduction

Forests play a very important role in the balance of the terrestrial carbon cycle [1,2]. They are, in general, net carbon sinks because the absorption of atmospheric CO_2_ is greater than that which is returned to the atmosphere through processes such as plant respiration and plant decomposition through bacteria, fungi and so on, thus making forest conservation of critical importance for mitigating climate change [3]. In this way, slowing deforestation, combined with an increase in forestation and other management measures to improve forest ecosystem productivity, could conserve or sequester significant quantities of carbon [4], thus fostering negative emissions and contributing to meet the Paris agreement aspiration of limiting global temperature rises to below 2 °C [5]. This sequestration of atmospheric carbon by forests also becomes a major strategy for the United Nations Framework Convention on Climate Change within the context of Reducing Emissions from Deforestation and forest Degradation (REDD), which could help mitigate greenhouse gas emissions on forest-rich developing countries [6], as is the case of Ecuador.

The economic and environmental relevance of natural and planted teak forests (*Tectona grandis* Linn. F.) is widely recognized. In 2010 the global area of planted teak forests reported from 38 countries was estimated at 4.35 million ha, which 83% grew in Asia, 11% in Africa, 6% in tropical America and less than 1% in Oceania [7], although taking into account the data missing from 22 teak growing countries, these figures could underestimate the actual planted teak forests in the world. Also according to this data, planted teak forests in Ecuador were around 45,000 ha in 2010, being this exotic species introduced more than 50 years ago in the province of Los Ríos, where it adapted and grew, becoming the main source of seeds for establishing commercial plantations [8]. In 2013, the Ecuadorian Ministry of Agriculture, Livestock, Aquaculture and Fisheries (MAGAP) recommended the establishment of pure teak plantations in coastal and Amazonian Ecuador within a governmental program of incentives for reforestation with commercial purposes, establishing a goal of 120000 ha of new planted teak forests in five years. However, progress in the suggested goals expressed as reforested area has been modest [8], although it is difficult to find reliable figures about the dynamic of new teak plantations due to a sub-registration and lack of updated inventories.

To help cope with the mentioned needs of efficient forests inventorying and monitoring, recent development in both active and passive remote sensing sensors has brought new types of spatial information and enhanced capacities to gain insight into emerging applications and methods especially suitable to be applied in the so-called Enhanced Forest Inventory (EFI). This approach to forest inventory makes use of advanced remote sensing technologies such as terrestrial laser scanning (TLS), airborne laser scanning (LiDAR; light detection and ranging) and digital aerial stereo-photogrammetry (DAP) based methods, in combination with ground plot data, to generate inventory attribute information [9]. In this sense, remote sensing is revolutionizing the way in which forests studies are conducted, and recent technological advances are providing new methods to measure aboveground biomass related variables from spaceborne, airborne and terrestrial sensors. However, to make full use of these emerging technologies for implementing forests inventories and quantifying forest carbon stocks, a new generation of allometric tools, based on individual tree measures that can be collected from spaceborne and airborne sensors (e.g., tree height and crown size), are needed [10]. In this regards, the recently emergence of computer vision based algorithms such as Structure from Motion (SfM) with Multi-View Stereo (MVS) [11] has boosted efficiency in collecting and processing geospatial information, yielding, under certain conditions, very dense and accurate 3D point clouds of comparable quality to existing laser-based methods [12,13,14]. This image-based approach [15] has demonstrated its potential to be successfully used in forest inventories [16,17,18,19], providing meaningful benefits to retrieve forest canopy structure, including reduced occlusions, high automation, low cost, limited manual editing, and increased geometric accuracy, when coupled with very-high resolution images acquired using Unmanned Aerial Vehicle (UAV) [20,21,22,23,24,25,26,27]. These new tools cannot be viewed as a logical progression of existing plot-based measurement, but a completely new way to face forest inventories. For example, TLS should be viewed as a disruptive technology that requires a rethink of vegetation surveys and their application across a wide range of disciplines [28].

Taking into account that accurate terrain extraction under canopy cover turns out to be crucial since most of the tree parameters involved in forest biomass estimates are related to the distance from the ground (e.g., diameter at breast height (DBH) and tree height) [29], the main objective of this work aims at developing and testing a SfM-MVS from UAV (UAV-DAP) based workflow to generate high quality Digital Terrain Models (DTMs) on teak plantations situated in the Coastal Region of Ecuador (dry tropical forest). To the best of the authors’ knowledge, this is the first study aimed at monitoring of teak plantations located in dry tropical forests from UAV images. 

Considering that some previous studies have demonstrated that DTMs from UAV images under leaf-off conditions achieved similar results to those of LiDAR [14,18,30], the underlying hypothesis that supports this work is based on the use of the alternating leaf-on and leaf-off phenological stages of teak trees associated with the rainy and drought seasons that characterize the climate of the dry tropical forest. In this sense, the leaf-on season would be used to obtain high quality canopy surface models (CSMs) from UAV images, while UAV images taken during the leaf-off season could potentially provide a suitable ground reference (DTM) to later build high quality canopy height models (CHMs) by subtracting the DTM from the CSM. The first-stage goal faced in this work, that is, obtaining accurate DTMs, constitutes a step forward to build the skeleton of an efficient remote sensing method to accurately estimate and update the aboveground biomass dynamic over small sample areas of the teak plantations located in the coastal region of Ecuador (i.e., at project level scale within the context of REDD). 

## 2. Materials and Methods

### 2.1. Study Area

**Figure 1:**
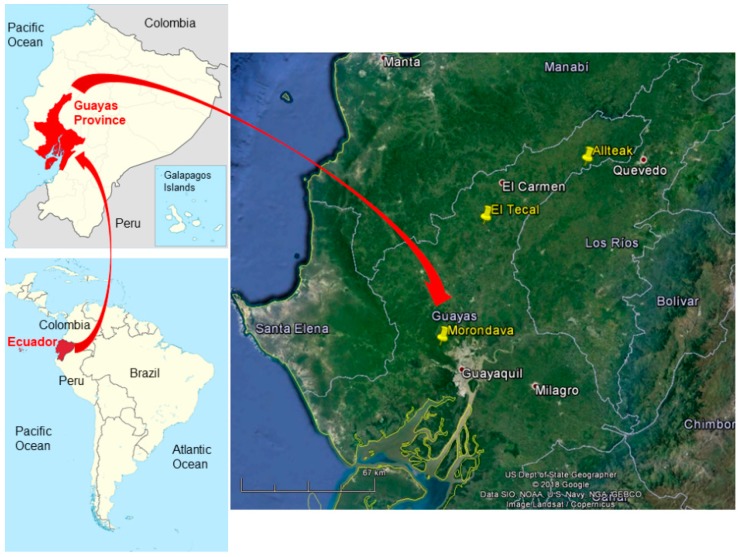
Situation map of the three teak plantations located in the province of Guayas (Ecuador): Morondava, El Tecal and Allteak.

The work area is located at the Coastal Region of Ecuador (Figure 1), comprising up to 58 planted teak (*Tectona grandis* Linn. F.) reference plots belonging to three different plantations: Morondava (2°6′11.72″ S, 80°2′59.43″ W), El Tecal (1°31′53.07″ S, 80°2′30.51″ W) and Allteak (1°8′7.23″ S, 79°41′58.08″ W).

The study site presents an average rainfall of about 1222 mm, with an average temperature of 24.4 °C and a relative humidity of 72.9% [31]. It belongs to the so-called Tropical Dry Forest [32], which is characterized by a very typical unimodal rainfall regime with a rainy period in the first quarter of the year and a marked drought during the rest of the year. These climate conditions cause alternating leaf-on (April to September) and leaf-off (November to December) seasons in the case of the teak plantations located at the coastal region of Ecuador, a climate-driven seasonal forest phenology which turns out to be very suitable to test the main hypothesis proposed in this work.

### 2.2. UAV-Based Photogrammetric Workflow and Point Cloud Generation 

The DJI Phantom 4 Advance^©^ (SZ DJI Technology Co., Ltd., Shenzhen, China) quadcopter UAV platform, carrying a 1” CMOS SENSOR (2.52 μm/pixel) with a 20 Megapixel resolution and 5472 × 3078 pixels fitted to a 3-axis stabilized gimbal to maintain nadir image capture, was used to take a very dense dataset of very high-resolution color red-green-blue (RGB) images over each one of the 58 reference plots. The area surveyed by the UAV over each plot presented an approximately square shape of 50 × 50 m, operating at a flying height of 50 m above ground level and using a focal length of 8.8 mm, which yielded an average ground sample distance of 1.4 cm (ground level reference). Each reference plot was treated as an independent UAV flight project because they were far away one each other. In this sense, 58 flight missions were accomplished. The field work was carried out between 8 and 23 November 2018, acquiring a high redundant set of overlapping images to avoid potential forest occlusions by working with forward and side overlaps higher than 80%. Five ground control points (GCP) constituted of rectangular wood panels (black and white chess-board style painted) were located approximately in the center and in the four corners of each reference plot (58 plots × 5 GCP per plot = 290 GCP in total), trying to choose open terrain sites to ensure their visibility in the UAV images. The WGS84 UTM 17S coordinates of the center of each photogrammetric panel were obtained by using a TOPCON ES105 total station. It was oriented for each reference plot from the observation of up to three GPS-RTK points, located very close to each reference plot, that were previously surveyed during a field campaign conducted between March and June 2018.

**Figure 2:**
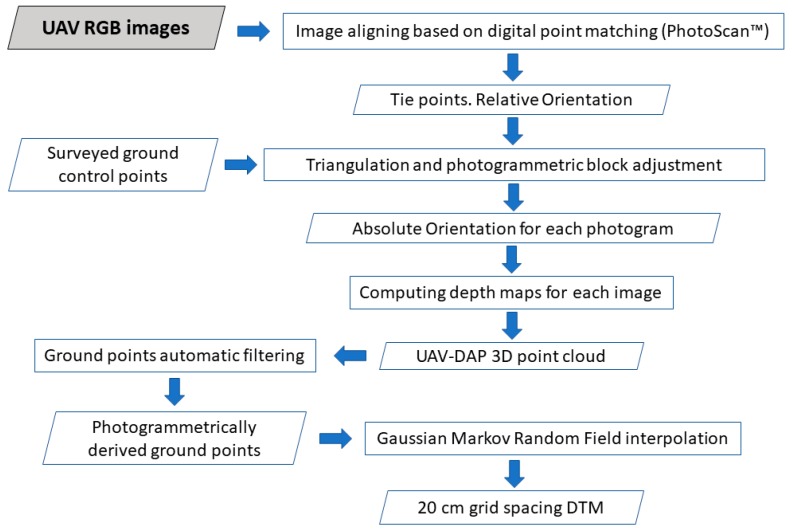
Flowchart representing the applied UAV-based photogrammetric workflow for 3D point cloud and grid DTM generation.

The pipeline shown in Figure 2 was applied to the UAV collected images in order to obtain a georeferenced UAV-DAP point cloud for each reference plot. The SfM-MVS algorithm implemented in the software Agisoft PhotoScan Professional Edition^©^ (Agisoft LLC, St. Petersburg, Russia) version 1.4.4 was used to undertake image alignment based on high density image matching [15], allowing to compute the camera position and orientation for each image and building a sparse point cloud model. PhotoScan is a well-known SfM-MVS software capable of producing high quality 3D point clouds from photogrammetric procedures [33], having being employed on numerous occasions to address UAV-based forest inventories [22,23,27,34].

The five surveyed GCP were manually marked on the digital images to carry out an iterative bundle adjustment to estimate the 3D coordinates (WGS84 UTM 17S) of the matched features [35]. A camera self-calibration process was performed within each reference plot to optimize the camera model by estimating principal point coordinates, affinity and skew transformation coefficients, and radial and tangential distortion coefficients. The focal length remained fixed during the self-calibration adjustment. After estimating internal and external camera orientation parameters, the depth information of each image was combined through a multi-view reconstruction of the scene geometry into a single and very dense 3D point cloud [35], rendering very high density UAV-DAP point clouds that ranged from 327 to 1006 points/m^2^ (average of 519 points/m^2^).

### 2.3. Point Cloud Filtering and DTM Generation 

Three sources of uncertainty must be kept under control when generating an accurate DTM: (i) Sample data error associated to building photogrammetrically derived point clouds), (ii) filtering error due to classifying ground points in non-open terrain, and (iii) interpolation or gridding error [36]. The first two sources are directly linked to the image-based point cloud generation described in Section 2.2, while the point cloud filtering and the DTM generation processes are addressed in this section.

Regarding point cloud filtering, the UAV-DAP point cloud generated by PhotoScan was automatically classified into ground and non-ground points (Figure 2) through applying the well-known filtering algorithm triangular irregular network (TIN) iterative approach proposed by [37]. This algorithm, implemented in PhotoScan Professional, divides point cloud data into square grids (cell size parameter) to find the seed points from local minima elevation. Those seeds are employed to build the reference TIN, iteratively adding candidate points to the TIN model representing bare earth points. Each candidate point is classified according to its distance to the nearest triangular surface and to the angle with the vertices of that triangle. The candidate point is classified as non-ground if the distance and angle are higher than predefined thresholds (distance and angle tuning parameters in PhotoScan). After a trial and error procedure, the parameters applied to all the reference plots were the following: cell size = 10 m, distance = 0.2 m, and angle = 15°. No manual editing of the point cloud was performed. 

Before converting the set of filtered ground points into a grid format DTM, potential outliers were automatically removed by adapting the parametric statistical method for error detection in digital elevation models published by Felicísimo [38]. This algorithm applies a parametric procedure based on the assumption that differences between the height of every point and its corresponding neighborhood mean height follows a normal distribution. In this case, the neighborhood size radius was established in each reference plot as five times the average grid spacing of the filtered ground points given by the square root of 1/GPD, being GPD the ground point density in points/m^2^. 

Finally, the scattered UAV-DAP derived ground points, potentially free of outliers, were interpolated to build a 20 cm grid spacing DTM comprising a square area of 36 m side by using the Gaussian Markov Random Field (GMRF) algorithm [39] (freely available code at https://github.com/3DLAB-UAL/dem-gmrf). 

### 2.4. Characteristics of the Reference Plots

Several dasometric variables were compiled at plot level for each one of the 58 reference plots of even-aged teak trees that take part in this study according to the following distribution: 30 plots were located in the Morondava teak plantation, 8 in the El Tecal plantation, and the remaining 20 in the Allteak plantation. A forest inventory field campaign was carried out by the Catholic University of Santiago de Guayaquil between the months of March to June 2018. Each tree within a sample plot was marked and numbered, recording its DBH with a measuring tape and other dendrometric variables such as crown diameter, total height and height to live crown for all trees within each plot using a laserAce™ 1000 (Trimble Navigation Limited, Westminster, CA, USA) rangefinder hypsometer [40]. Some dasometric variables, especially related to the structure of the forest and its impact on the visibility of bare earth from a top view (UAV-DAP), were derived from the dendrometric variables registered in each reference plot. The dasometric variables considered in this work were tree density (trees/ha), basal area per hectare (G; m^2^/ha) given by the sum of the cross-sectional areas of tree trunks at 1.3 m in height, and Lorey’s mean height for each reference plot computed as a weighted mean height whereby individual tree heights are weighted in proportion to their basal area. It is worth noting that Lorey’s mean height has been often used as the ground truth of mean tree height of forest stands in remote sensing studies [41].

Other morphological variables computed at reference plot level were mean slope and estimated vegetation coverage. The mean slope (%) for each plot was estimated as the maximum difference in height between the points of the corresponding DTM (Section 2.3) divided by the side of the square plot (36 m). The estimated vegetation coverage for each plot was computed from the UAV-DAP point cloud as the percentage of the non-ground points (likely vegetation points) classified through the TIN iterative approach algorithm (Section 2.3) over the total number of UAV-DAP derived points (i.e., ground and non-ground classified points). This variable could be taken as an estimate of canopy density that hampers the visibility of bare ground from images taken from UAV aircrafts [16,20]. 

### 2.5. TLS-Based Ground Truth

**Figure 3:**
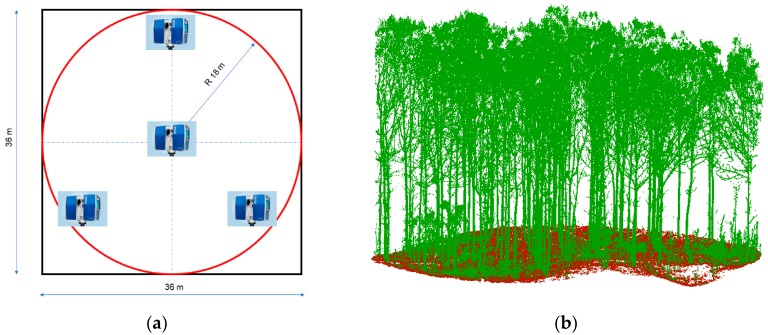
Reference plots geometry and TLS-derived point cloud. (**a**) Square and circular reference plots for UAV-DAP derived DTM and TLS field work, respectively. The four scans pattern is shown; (**b**) Semi-automatically segmented TLS point cloud showing Ground (brown) and Vegetation (green) classified points. Case study: reference plot number 1 (El Tecal plantation).

TLS has proven to be an effective technique for both extracting specific tree attributes in forest plots (tree position, tree height, DBH, stem curve, etc.) [29] and building DTM beneath forest canopy [42]. In this work, a very dense and accurate TLS point cloud was obtained within each reference plot simultaneously to the UAV flights to be used, after proper processing, as a DTM ground truth. The TLS point clouds were captured through a field survey carried out with a FARO Focus 3D X-330 TLS instrument able to record millions of 3D points along a range from 0.6 to 330 m and a vertical and horizontal field of view of 300° and 360°, respectively. Its nominal distance accuracy is around ±2 mm. Four scanning positions were set up within a radius of 18 m from the center of each reference plot to configure a scan pattern with a central scan and the rest located around drawing an equilateral triangle (Figure 3a).

The FARO Scene^©^ software was utilized to co-register the four scans within each reference plot, thus producing a single TLS point cloud, from using nine artificial targets (15 cm diameter spheres) conveniently distributed over the reference plot to ensure that at least three spheres were visible from every two consecutive scan positions. The mean distance error of the co-registered point clouds for the 58 reference plots was 6.2 mm, ranging from 2 mm to 13.3 mm. The scan located at the center of each reference plot (reference scan for the co-registering process) was georeferenced by applying a 3D conformal coordinate transformation based on the WGS84 UTM 17S coordinates of 4 spheres (out of the nine available) surveyed by using a TOPCON ES105 total station previously oriented as described in Section 2.2.

A very accurate scattered bare earth points were obtained from automatically segmenting TLS ground points by means of the octree search algorithm implemented in the open-source software 3D Forest [29] (Figure 3b). This algorithm recursively subdivides the 3D space of the TLS point cloud into eight cubes until arriving at the specified resolution R (length of the cube edge). A set of local minima are extracted by segmenting the lowest point on the Z-axis based on a search in the octree structure. A two-pass processing, increasing cubes resolution from 10R to R, is undertaken to minimize noise points. The lowest points of the lowest cubes are labelled as ground points. In this case some manual edition was performed to ensure a high accuracy in the extracted ground points, since this dataset was employed as ground truth for the vertical accuracy assessment of the UAV-DAP derived DTM obtained as described in Section 2.2 and Section 2.3.

### 2.6. DTM Accuracy Assessment

The vertical accuracy of the UAV-DAP derived DTM for each reference plot was assessed by computing the vertical difference between the Z value in the TLS-derived scattered ground points (ground truth) and the corresponding bilinearly interpolated Z value extracted from the 20 cm grid spacing UAV-DAP DTM. After removing blunder errors from the signed z-differences by applying the widely known three-sigma rule [43], vertical accuracy statistics such as mean, median, standard deviation, maximum value, minimum value and 90th (LE90) percentile linear error were computed to estimate the vertical accuracy of the UAV-DAP DTM generated in each reference plot [44].

A four-way ANOVA (Equation (1)) was applied to investigate the variability and relationship of some plot-level features on DTM accuracy. The dependent variable for each analysis was an estimate of the random and systematic vertical errors (Sd, L90 and mean value) of the UAV-DAP derived DTMs. The independent categorical variables were features computed at plot-level such as the basal area (G), Lorey’s mean height (Lh), estimated vegetation coverage (VC) and mean slope (MS). Both VC and MS were estimated from the UAV-DAP derived point clouds. The plots were grouped into four arbitrary defined classes for each studied plot-level feature according to the following intervals: G classes {[0–5] (5–10] (10–15] and >15 m^2^/ha}; Lh classes: {[0–8] (8–13] (13–20] and >20 m}; VC classes {[0–20] (20–40] (40–60] and >60%}; and MS classes {[0–12] (12–24] (24–40] and >40%}. Significant differences (*p* < 0.05) between classes regarding DTM accuracy statistics was accomplished using an unequal Tukey’s HSD. This test is a generalization of Tukey’s test to the case of unequal or unbalanced samples sizes [45].

X = Intercept + αG + βLh + γMS + δVC + ε(1)

## 3. Results

### 3.1. Characteristics of the Reference Plots

Some characteristics of the reference plots that take part in this study are presented in this section. Most of the features are related to the spatial arrangement and density of the teak trees located within each sample plot because the presence of dense canopy reduces the performance of airborne sensors to provide measurements from the canopy through to the ground surface [46]. This effect of occlusion is especially significant in the case of image-based method such as UAV-DAP [17]. The key features of the reference plots located in the plantations of Morondava, El Tecal and Allteak are shown in Table 1, Table 2 and Table 3, respectively. 

**Figure 4:**
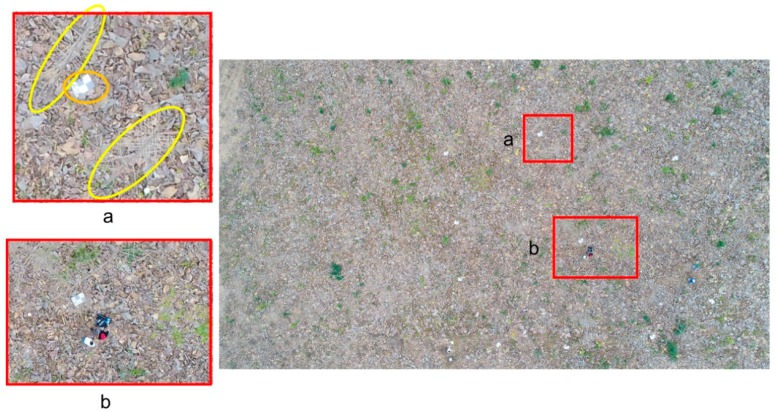
Example of an RGB original image taken at a flying height of 50 m (AGL) over the reference plot number 2 (Morondava). Estimated vegetation coverage = 0%. Note in the zoom-in area “a” the completely leaf-off stage of the teak trees (yellow ellipses showing trees inclined due to the perspective view), with a large amount of fallen leaves on the ground, and a detail of a rectangular panel (orange ellipse) used to mark the GCPs. Also note the small size of the trees compared to the two people who are setting one of the TLS scanning positions in the zoom-in area “b”.

Morondava would be a good representative of a typical young teak plantation with plantation ages between two and three years and an average planting density (surviving trees) of 722 trees/ha. This is a very early developing plantation that shows a certain heterogeneity with both a low average basal area of 4 m^2^/ha (from 0.6 to 6.28 m^2^/ha) and a low average Lorey’s mean height of 7.83 m (from 3.84 to 9.57 m). Considering that both basal area [47] and Lorey’s mean height [48] are dasometric variables positively correlated to forest stand volume, a poor canopy presence can be inferred in the reference plots located in the Morondava plantation. This point is partially confirmed by the low estimates of vegetation coverage (VC) shown in Table 1. In fact, the average VC took a very low value of 4.47%, although some reference plots presented higher VC values of up to 33.65% (plot number 25). Note that although the UAV fieldwork was carried out in the dry season of the coastal region of Ecuador (i.e., leaf-off phenological stage), some plots still contained trees presenting usually dry leaves that had not yet fallen to the ground. This was because they were located in areas with greater soil water availability due to their edaphological and/or geomorphological characteristics (e.g., they were situated in depressions or troughs where rainfall water accumulates). In any case, most of the reference plots belonging to the Morondava plantation seemed to have an almost transparent canopy from a top view, as can be seen in Figure 4, meaning that the SfM algorithm is not able to extract canopy points over teak plantations when leaves are poor or absent. 

Regarding El Tecal, it is a 17-year-old plantation much more developed than Morondava and with an average planting density of 985 trees/ha (Table 2). Both basal area and Lorey’s mean height presented average values of 16.78 m^2^/ha and 15.36 m, respectively, significantly higher than those registered in Morondava. In this sense, it can be deduced that the reference plots located in El Tecal presented a forest stand volume larger than those of Morondava. However, the average VC (Table 2) in El Tecal turned out to be 1.84%, an even lower value than the one registered in Morondava, indicating that a greater volume of forest stand is not directly related to the percentage of canopy points that the SfM algorithm is capable of extracting. In fact, VC seems to be more related to the presence of leaves (dry or green) than to the volume of forest stand. It is necessary to highlight a low presence of leaves in the teak trees of El Tecal plantation, likely due to its high planting density and low soil water availability.

In Table 3 are depicted the main characteristics of the reference plots located in the Allteak plantation. The age of plantation varied between 4 and 12 years, showing a very heterogeneous planting density with an average of 579 trees/ha, a value lower than that registered in Morondava or El Tecal due to the usual application of thinning operations. Basal area values ranged from 5.17 to 17.12 m^2^/ha (average of 11.56 m^2^/ha), while Lorey’s mean height values varied from 12.30 m to 23.02 m (average of 18.57 m).

**Figure 5:**
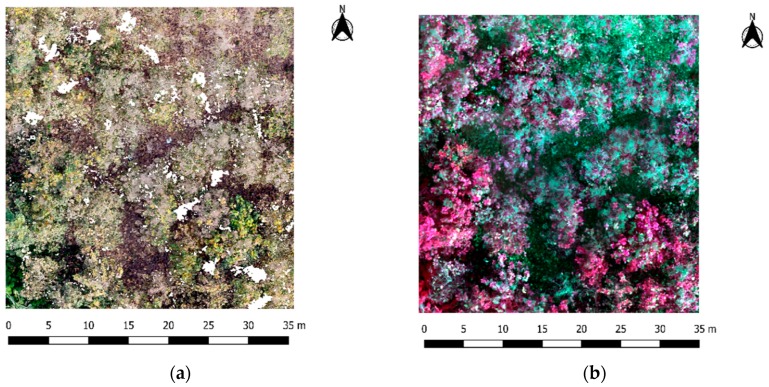
Orthoimages (5 cm ground pixel size) computed for the reference plot number 13 (Allteak). Estimated vegetation coverage = 83.96%. (**a**) True color composite (R-G-B) orthoimage. (**b**) Nir false color composite (Nir-R-G) orthoimage composed of images taken from a Parrot Sequoia© (Parrot SA, Paris, France) multispectral sensor on-board the UAV DJI Phantom 4 Advance© used in this work.

It is important to underline that tree height growth and tree architecture is influenced by both endogenous features and exogeneous factors (competition for light, water and nutrients) [49,50]. These exogeneous factors are clearly related to planting density, age and silvicultural management, which can explain the reason why the trees located in the Allteak plantation present a higher primary tree growth (growth in height estimated from Lorey’s mean height) than secondary growth (related to basal area) as compared to the trees belonging to El Tecal plantation. The low tree density, in addition to a greater soil water availability due to a better edaphological soil conditions and the presence of numerous water streams in the Allteak plantation, can also explain its large average VC of 26.49%, with some reference plots reaching values above 80% (see Figure 5a) because of the presence of abundant, usually, dry leaves. The greater presence of dry leaves with respect to green ones can be seen in Figure 5b, that shows a Nir false color composite (Nir-R-G) orthoimage where red color indicates photosynthetically active leaves. 

### 3.2. Vertical Accuracy of UAV-DAP Derived DTM

**Figure 6:**
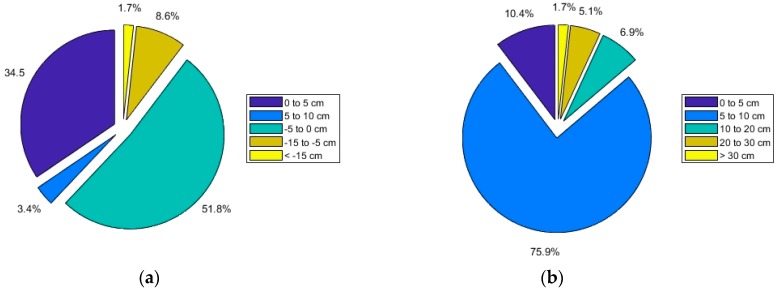
Exploded pie diagrams of the distribution of mean values (**a**) and standard deviations (**b**) computed from the DTM vertical errors in the 58 reference plots.

In Table 4 are depicted some meaningful vertical error statistics computed over the 58 sample plots available. Both the mean and median statistics provide a picture about the DTM bias or systematic error. Therefore, average values of −3.1 cm and −3.24 cm for the mean and median DTM vertical error over the 58 reference plots should be interpreted as suitable to support the generation of accurate CHMs in teak plantations inventories. The slight bias found in the z-differences distribution (negative values) points to the fact that the UAV-DAP DTM tended to slightly overestimates the reference ground elevation, a phenomenon that is also frequently encountered when working with LiDAR data in afforested areas due to dense low lying vegetation [51,52,53]. Here, it is worthy to note that 62.1% of the reference plots yielded negative DTM bias (Figure 6a). However, excessively large and unacceptable negative DTM bias of up to −130.9 cm (mean vertical error) was assessed in the reference plot number 14 located in the Allteak plantation. This plot registered an extremely high VC value of 90.93%. In the rest of the reference plots, the mean vertical error remained below 15 cm in terms of absolute values (Figure 6a). Moreover, up to 86.3% of the reference plots presented DTM mean vertical error lower than 5 cm (absolute values), as can be also observed in Figure 6a.

On the other hand, the UAV-DAP DTM vertical random error was greater than the systematic error, as can be deduced from the average value of standard deviation (SD) of z-differences (Table 4). An average SD value of 11.9 cm and up to 86.3% and 93.2% of the reference plots showing SD values below 10 cm and 20 cm, respectively (Figure 6b), can be considered as sufficiently accurate results to support the realization of derived teak plantation inventories [54]. The same can be said of 90th percentile vertical error (LE90), presenting an average value of 21.4 cm which means that 90% of the z-differences were below this figure. It was observed that extremely high maximum values of 228.6 cm and 444.1 cm for SD and L90 statistics were registered in the reference plot number 14 located in the Allteak plantation, that was the plot with the highest VC value (see Figure 8).

**Figure 7:**
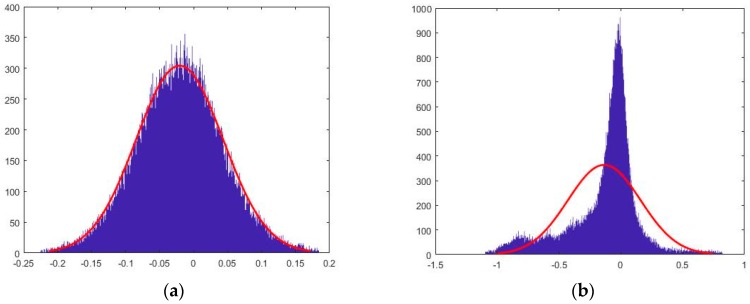
Z-differences distribution at two different reference plots. The corresponding normal distribution is overlaid in red. (**a**) Reference plot number 2 (Morondava plantation). (**b**) Reference plot number 13 (Allteak plantation). Units of horizontal axis in meters.

The maximum and minimum vertical error registered at plot level over the 58 sample plots took values of 493.5 cm and −674.9 cm, respectively (Table 4), although with maximum (36.8 cm) and minimum (−40.2 cm) average values reasonably low, thus pointing to the presence of some punctual outliers that could not be conveniently removed by the automatic algorithm described in Section 2.3. In any case, the z-differences distribution in most of the reference plots that had low VC values (e.g., Morondava plantation) followed an almost perfect normal distribution (Figure 7a), whilst clearly non-normal distributions were found in reference plots with VC values higher than 50% (Figure 7b).

### 3.3. Relationship between the Vertical Accuracy of UAV-DAP Derived DTM and Reference Plots Features

The four-way ANOVA of the plot-level features examined (sources of variation) allowed to check their significant influence (*p* < 0.05) on the UAV-DAP DTM vertical error. As depicted in Table 5, Table 6 and Table 7, the estimated vegetation coverage turned out to be the source of variation that could explain most of the model’s global variance for both the systematic error (mean value, Table 5) and the random error (SD and L90; Table 6 and Table 7, respectively). In fact, it presented a significant influence in all the vertical error statistics evaluated, explaining 26% of the variability of the mean error, 30.68% of the random error variability based on SD and 30.23% of the variability of L90.

The other significant source of variation was basal area, a dasometric variable positively correlated to stand volume [47]. Although basal area only explained between 6.9% (SD of z-differences variability at plot-level) and 8.8% (mean of z-differences variability at plot-level), their scores were found statistically significant at a significance level α = 0.05.

Neither Lorey’s mean height nor plot mean slope were found to significantly explain the variability of the DTM vertical error statistics computed in this work. Particularly, the average slope of the plot proved to have a very small influence on the variability of the UAV-DAP DTM vertical accuracy, only explaining less than 1% of the overall variance of the model. It will be discussed in the corresponding section. 

In Table 8 are shown the statistical results corresponding to the means separation between groups for all the sources of variation tested. Despite not having been found significant in the previous four-way ANOVA, the Lorey’s mean height and mean slope sources of variation have been included for the sake of clarity. 

Within the estimated vegetation coverage feature, we can underline that only the group of plots with VC > 60% was pointed as showing DTM vertical accuracy statistics significantly different with respect to the other three groups. Regarding mean vertical error at plot-level, the reference plots with VC > 60% overestimated the ground reference provided by the TLS survey by approximately 45 cm on average. The average SD and L90 plot-level values recorded in the VC > 60% group were significantly higher than those computed in the other three groups.

No significant differences were found between the four groups defined from the variable basal area, despite the significant results yielded by the ANOVA test. Note that the Tukey’s test was adjusted to deal with unequal samples sizes, making it more difficult to achieve statistically significant results. 

**Figure 8:**
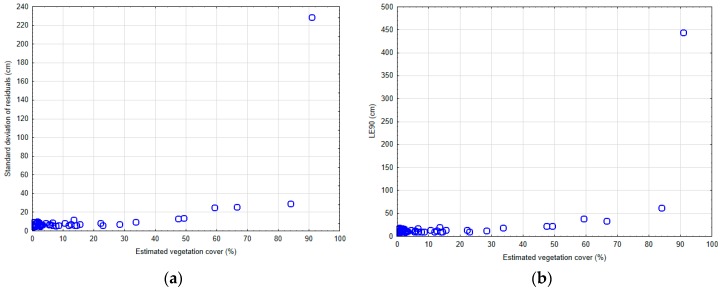
Relationship between estimated vegetation coverage and vertical random error of UAV-DAP DTM. (**a**) Standard deviation of DTM z-differences. (**b**) 90th (LE90) percentile error of DTM z-differences.

The relationship between the estimated vegetation coverage and the DTM random error, given by SD and L90 statistics, is plotted in Figure 8. As expected, the behavior of both estimates of random error in relation to the variation of VC was similar, presenting a gradual increase of values around 50% forward and almost exponential for values above 90%. In this sense, it is worth pointing out that practically all the reference plots showed an acceptable random error of less than 30 cm, except plot number 14 (Allteak plantation), which presented extremely high values of more than 2 m of standard deviation associated with large values of up to more than 90% of the estimated vegetation coverage.

## 4. Discussion

Because the spatial distribution of canopy heights is usually computed by subtracting a DTM from the elevation of the digital surface model (DSM) of the outer canopy layer, the quality of the canopy height estimates is closely correlated with DTM quality [51]. In this way, the generation of a high quality DTM is one of the pillars of a UAV-based method for inventorying teak plantations in tropical dry forests such as those located in the coastal region of Ecuador.

However, low DTM completeness is especially common when dealing with remote sensing forestry applications, where data acquisition (e.g., laser beam penetration through canopy in LiDAR surveys) can be limited and so the ground sampling density is consequently reduced [36]. In the main, LiDAR can penetrate the forest canopy providing scattered ground points to support DTM interpolation, while UAV-DAP is limited to the production of DSMs because imagery only provides measures for the canopy surface as visible from the air [23,34,55]. In this regards, ground elevation error in LiDAR derived DTMs are usually less than 30 cm under forest covers [56,57], although it can vary a lot depending on both vegetation structure and density [57]. In addition, LiDAR surveys are sometimes unaffordable in developing countries [25], with DAP estimated to be one-third to one-half the cost of LiDAR data for inventorying the same forest area [9]. 

Being aware of the limitations of UAV-DAP technology to generate high quality DTMs when the surveyed area presents dense vegetation [23,25], we propose in this work to place the date of UAV imagery acquisition at the end of the dry season in the coastal region of Ecuador (i.e., from November to December), that is coinciding with the leaf-off phenological stage of the teak plantations. Acquiring UAV images during leaf-off season can meaningfully diminish ground surface visual occlusions due to canopy, so improving SfM-MVS point matching process and, consequently, the accuracy of UAV-DAP derived DTM [58]. Complementary UAV field work should be faced at the end of the rainy season (leaf-on) to obtain high quality CHMs given by the difference between CSM (leaf-on capture) and DTM (leaf-off capture). A similar strategy was successfully tested by [18] working on temperate deciduous forest sites in Maryland (USA). In this case, understory DTMs and CHMs were generated from leaf-on and leaf-off UAV-DAP derived point clouds using procedures commonly applied to LIDAR point clouds. In the same way, Moudrý et al. [14] reported that a proper combination of leaf-off and leaf-on UAV imagery could have the potential to replace LiDAR data, pointing to leaf-off UAV imagery as a viable alternative for building DTMs. In other very recent work, Moudrý et al. [30] successfully identified the bare ground during the leaf-off period in a deciduous forest using images from two different fixed-wing UAV systems.

According to this strategy, the results achieved in this preliminary first stage, i.e., UAV imagery capture over teak plantations in leaf-off season, can be considered as very promising, obtaining UAV-DAP DTM average vertical error estimates (in terms of SD) of 11.9 cm, along with an average systematic error close to zero (−3.1 cm) (Table 4). It is relevant to bear in mind that the SD of z-differences were kept below 20 cm in 93.2% of the reference plots and below 30 cm in all the plots, except in the plot number 14 located in the Allteak plantation (Figure 6b). Note that these figures are not very far from those usually provided by the standard and existing UAV terrain mapping algorithms and software, which are able to produce very accurate and reliable DTMs (mean vertical error = −1.9 cm and RMSE = 3.5 cm) over exposed bare grounds [59].

The results obtained in this work were more accurate than those reported by [18,22,25,60,61], mainly because all the referred research works were undertaken over closed leaf-on canopy forest stands. For example, Jensen et al. [22] applied UAV-SfM derived point clouds to build under vegetation DTMs in fifteen 20 × 20 m plots, reporting a mean vertical error of −0.19 m (ground reference overestimation) and a SD value of 0.66 m. Mlambo et al. [25] found a DTM vertical error of up to 2.31 m (RMSE) working with UAV images and VisualSfM open source software over a very closed canopy forest plot (2.3 ha) that comprised dominant Sycamore (*Acer pseudoplatanus*) and Scots pine (*Pinus sylvestris*), while Yilmaz et al. [61] recorded DTM vertical error of 30 cm and 28.7 cm (SD of z-differences), and 15.8 cm and −16.6 cm (mean z-differences), from using images taken by a fixed wing UAV over two test sites presenting relatively closed canopy forest stands. Finally, Ota et al. [60] quantitatively proved that a more accurate DTM than the DTM derived from aerial photographs using the SfM approach would be needed to accurately estimate aboveground biomass in the case of closed canopy leaf-on tropical forests. 

On the other hand, Moudrý et al. [30] reported vertical root mean square error (RMSE) values ranging between 0.11 and 0.19 m from understory UAV-DAP DTMs generated in a deciduous forest under leaf-off conditions, while Moudrý et al. [14] also found that the vertical accuracy of image-based DTMs declined in the following order: Forest under leaf-off conditions (RMSE 0.15 m), steppes (RMSE 0.21 m), and aquatic vegetation (RMSE 0.36 m). Aguilar et al. [27] obtained low vertical error (SD = 7.4 cm), although showing higher bias (mean z-differences = −10.4 cm), by applying a workflow similar to the one used in this work over a 50 × 50 m plot located in a typical Mediterranean forest composed of an upper layer of Aleppo pine (*Pinus halepensis Mill.*) and understory vegetation mainly formed of little holm oak trees (*Quercus ilex L.*) and different species of shrubs. The total canopy cover was estimated at about 54.5% in this case. Similarly, Wallace et al. [23] found that both UAV-DAP SfM and LiDAR provided a good representation of the terrain (mean z-differences = −9 cm) working over a 30 × 50 m plot located in a dry sclerophyll eucalyptus forest with spatially varying canopy cover, although the authors also underlined that the terrain was not adequately sampled in the SfM point cloud within under canopy areas. In fact, this is one of the key factors that explains the remarkable results attained in our study. As can be made out in Figure 8, the UAV-DAP SfM technique was able to correctly model the under-canopy terrain in the case of leaf-off teak plantations with VC < 60%. VC values higher than 60% at the plot level were associated with less accurate UAV-DAP DTM, as revealed in the ANOVA test shown in Table 5, Table 6 and Table 7. Similar results are reported by [25], where SfM from UAVs proved to perform poorly in closed canopies, though providing suitable results in areas with sparse canopy cover (<50%).

The second key factor that can help explain the accurate DTMs obtained in this work would be the high accuracy photogrammetric bundle adjustment achieved when computing the internal and external camera orientation parameters. The rigorous field topography campaign conducted to georeference the five GCPs within each reference plot and the good performance of the SfM-MVS algorithm over the teak plantations in leaf-off conditions, resulted in an average point cloud planimetric and vertical error of 1.6 cm (maximum value of 6.4 cm) and 1.2 cm (maximum value of 5.5 cm), respectively (object space error). Regarding image space error, an average re-projection error of 0.48 pixels (maximum value of 0.91 pixels) was achieved. In any case, there is a growing need to explore the impacts of different image acquisition and processing parameters on DAP SfM derived outputs such as flying height, software package choice and parameter settings [60,62,63].

The last key factor that supports the accurate results reached in this work would be the good performance of both the ground points filtering algorithm (including outlier removal) and the interpolation method applied to generate the final DTM. Even though the point cloud filtering results could have been better if the three TIN iterative approach parameters had been properly tuned for optimum performance in each reference plot, we preferred to keep them constant and adopt conservative values in order to speed up the process and ensure a low number of false positives (type I error). Furthermore, the TIN iterative approach has demonstrated to be one of the most robust point cloud filtering algorithms [64]. The automatic outlier removal applied just before building the DTM also helped to ensure the reliability of the points labeled as ground.

As previously explained, it is usually necessary to densify the initial UAV-DAP point cloud when the surveyed area presents dense vegetation. The new ground points must be interpolated to infill the gaps and construct accurate DTMs. Interpolation methods used for infilling gaps may produce a non-negligible error usually named gridding error [65], which is due to the propagation of the sample data error (point cloud error in this case) towards interpolated points. In this sense, gridding error depends on sample data error, initial sample point density, terrain complexity and interpolation method [66,67]. In this work, the terrain complexity has been estimated using the average slope of the plot as a terrain descriptor, not resulting in a plot feature significantly correlated with the vertical DTM error, as can be seen in the ANOVA tests that are shown in Table 5, Table 6 and Table 7. Note that a well-known characteristic of observed elevation error for terrain mapping is the relationship with terrain slope, especially in the case of DTM generation by means of laser scanning, where planimetric error may be relatively high and also may be directly translated to vertical error on sloping surfaces [36,68]. However, the planimetric error of the UAV-DAP point clouds computed in this work took a value of 1.6 cm on average, much lower than the nominal planimetric error of LiDAR derived point clouds. Moreover, the terrain average slope turns out to be not suitable to explain full terrain complexity in some occasions [69].

**Figure 9:**
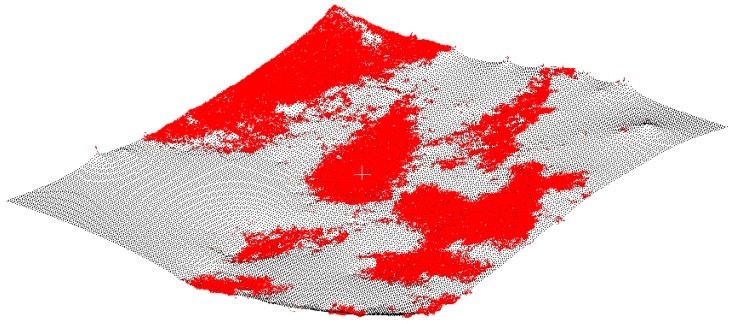
Perspective block diagram of the original photogrammetrically derived ground points (red) and the corresponding DTM interpolation (mesh in black) for the reference plot number 13 (Allteak plantation). Estimated vegetation coverage = 83.96%.

In Figure 9 are depicted the UAV-DAP points labelled as ground (in red) overlaid onto the GMRF interpolated DTM in the case of the reference plot number 13 (Allteak) in which the estimated VC reached a large value of 83.96%. In this respect, it can be highlighted the abundance of ground gaps to be interpolated in order to obtain a continuous surface for modelling the terrain topography under forest canopy. Despite this fact, the GMRF interpolation algorithm was able to properly fill the ground blanks yet producing a smooth and apparently truthful DTM. Furthermore, GMRF does not require to specify the local support or kernel (searching radius or maximum number of neighbors intervening in the interpolation of each grid point), which can be qualified as very advantageous, above all when dealing with low density ground points areas such as forest environments [39].

## 5. Conclusions

In order to obtain high quality DTMs as ancillary data to support efficient teak plantations inventories in the tropical dry forest located in the coastal region of Ecuador, and considering that most of the teak plantations are managed under rainfed conditions, the results obtained in this work have confirmed the initial hypothesis pointing to the convenience of carrying out the UAV imagery acquisition at the end of the dry season (i.e., from November to December) when teak trees are practically leafless. This strategy has proven to be suitable to increase the exposed bare ground by avoiding ground surface visual occlusions from a top view (UAV-DAP) due to presence of dense canopy, thus improving SfM-MVS point matching at ground level and, consequently, the accuracy of the UAV-DAP derived DTM. Complementary UAV field work should be faced at the end of the rainy season (leaf-on phenological stage), i.e., from approximately April to May in the climate conditions or our study site, to obtain high quality CHMs given by the difference between CSM (leaf-on capture) and DTM (leaf-off capture).

The mentioned strategy resulted in UAV-DAP derived DTMs presenting low bias or systematic error, with an average plot-level value of −3.1 cm for the mean vertical error calculated on the 58 reference plots that took part in this study. It means that the UAV-DAP DTM tended to slightly overestimates the reference ground elevation, although is quite remarkable that up to 86.3% of the reference plots presented DTM mean vertical error lower than 5 cm in terms of absolute values. 

Regarding vertical random error, the average SD value the UAV-DAP derived DTMs was greater than the mean vertical error, taking an average value of 11.9 cm. Here it is worth noting than up to 93.2% of the reference plots showed SD values below 20 cm, which can be considered as a reasonable vertical error threshold as compared to the accuracy provided by LiDAR based methods.

Both basal area and estimated vegetation coverage, two plot-level features closely related to forest stand volume and structure that may affect the visibility of bare earth from a top view (UAV-DAP), presented a statistically significant (*p* < 0.05) relationship with the tested plot-level DTM vertical error statistics such as mean, SD and L90. In fact, the higher the basal area or the estimated vegetation coverage, the higher the vertical error in the DTM derived from UAV-DAP. This relationship was particularly meaningful from threshold values above 15 m^2^/ha and 60% in the cases of basal area and estimated vegetation coverage, respectively. 

The high quality UAV-DAP derived DTMs obtained in this work, together with the corresponding UAV-DAP derived CSMs that will be generated in leaf-on conditions and the allometric equations which are being developed in the context of the research project that finances this study, constitute the basis of an efficient remote sensing based method to carry out an upscaling process headed up to estimate aboveground biomass over the teak plantations located in the coastal region of Ecuador. This method could potentially be applied, after proper calibration and validation, to other teak plantations located in seasonally dry tropical forests to assist in the REDD monitoring and sustainable management.

## Figures and Tables

**Table 1:** Characteristics of the reference plots located at the teak plantation of Morondava.

Plot Number	Age of Plantation	Density (trees/ha)	Basal Area (m^2^/ha)	Lorey’s Height (m)	Mean Slope (%)	Vegetation Coverage (%)
1	3	680	4.87	8.90	4.60	0.04
2	3	860	5.86	9.08	25.64	0.00
3	3	720	3.63	8.42	17.98	0.04
4	3	820	5.10	8.55	22.81	0.01
5	3	800	5.13	8.37	15.75	0.03
6	3	780	6.01	8.97	9.44	0.03
7	3	660	4.80	8.86	22.88	0.54
8	3	540	2.29	7.16	3.94	0.03
9	3	660	2.90	8.91	20.59	0.67
10	3	860	6.10	9.38	31.82	0.79
11	3	760	4.41	9.27	34.59	0.28
12	3	600	3.52	8.63	7.14	0.83
13	3	680	4.13	8.35	29.39	28.32
14	3	780	5.26	7.97	5.61	6.48
15	3	760	2.80	6.83	18.69	22.83
16	3	660	4.65	8.93	16.16	2.52
17	3	600	4.32	9.57	12.84	2.97
18	3	840	4.79	7.99	17.91	2.56
19	3	820	4.00	7.23	16.68	2.24
20	3	700	2.60	6.57	43.05	1.66
21	3	260	0.60	4.89	9.14	0.77
22	3	820	5.35	8.40	11.73	1.88
23	3	780	2.23	5.32	43.96	1.67
24	3	740	5.24	8.29	37.83	2.01
25	3	740	4.90	9.20	42.91	33.65
26	3	720	4.65	9.33	36.92	1.54
27	2	660	1.07	4.15	12.13	10.60
28	2	820	1.62	4.58	41.80	6.47
29	3	700	0.84	3.84	55.54	0.45
30	3	840	6.28	9.01	33.67	2.31
Average	2.93	722	4.00	7.83	23.43	4.47
SD ^1^	0.25	120.38	1.61	1.67	13.92	8.51

^1^ Standard deviation.

**Table 2:** Characteristics of the reference plots located at the teak plantation of El Tecal.

Plot Number	Age of Plantation	Density (trees/ha)	Basal Area (m^2^/ha)	Lorey’s Height (m)	Mean Slope (%)	Vegetation Coverage (%)
1	17	700	15.81	15.78	10.27	2.47
2	17	780	17.08	16.84	11.16	0.21
3	17	760	19.25	19.20	12.54	0.38
4	17	860	17.49	17.32	11.23	1.07
5	17	1480	19.58	13.66	12.37	4.31
6	17	1220	18.43	14.96	13.55	0.03
7	17	1020	14.26	14.00	14.74	5.43
8	17	1060	12.37	11.12	8.32	0.84
Average	17	985	16.78	15.36	11.77	1.84
SD ^1^	0	266.35	2.50	2.50	1.99	2.03

^1^ Standard deviation.

**Table 3:** Characteristics of the reference plots located at the teak plantation of Allteak.

Plot Number	Age of Plantation	Density (trees/ha)	Basal Area (m^2^/ha)	Lorey’s Height (m)	Mean Slope (%)	Vegetation Coverage (%)
1	12	780	11.16	17.86	20.66	12.47
2	12	940	9.91	20.36	19.05	15.45
3	12	640	12.26	13.04	14.08	66.50
4	12	300	14.33	21.45	14.61	13.76
5	12	500	14.23	21.47	4.25	1.19
6	12	360	13.25	21.44	17.76	59.24
7	12	420	13.23	18.04	19.52	5.66
8	12	420	12.90	23.02	12.28	49.22
9	10	340	14.07	22.14	43.93	47.40
10	4	420	8.95	13.13	33.17	11.85
11	12	620	5.17	14.58	6.83	13.52
12	12	740	13.45	20.82	8.50	8.56
13	4	580	12.97	15.28	40.79	83.96
14	4	560	17.12	16.72	23.25	90.93
15	12	780	9.52	20.26	4.47	7.70
16	12	680	8.53	19.55	3.96	3.35
17	12	560	7.68	20.14	2.53	0.85
18	12	620	12.35	22.09	3.11	1.75
19	4	620	7.82	12.30	27.51	22.22
20	12	700	12.33	17.80	16.66	14.32
Average	10.30	579	11.56	18.57	16.84	26.49
SD ^1^	3.26	168.01	2.89	3.36	12.20	28.71

^1^ Standard deviation.

**Table 4:** Range of variation of the DTM z-differences (Z_TLS_ − Z_UAV-DAP_) statistics computed at reference plot level (number of observations = 58 plots). All figures are expressed in cm.

Statistics Computed at Plot Level	Average Value and Standard Deviation (between Brackets) for All Plots	Range of Variation (Minimum to Maximum Values)
Mean	−3.1 (17.4)	−130.9 to 8.9
Sd ^1^	11.9 (29.3)	4.4 to 228.6
Median	−3.2 (17.4)	−132.3 to 5.7
Maximum	36.8 (63.9)	13.8 to 493.5
Minimum	−40.2 (86.7)	−674.9 to −9.7
LE90 ^2^	21.4 (57.1)	7.9 to 444.1

^1^ Standard deviation. ^2^ 90th percentile vertical error.

**Table 5:** Four-way ANOVA of basal area (G), Lorey’s mean height (Lh), vegetation coverage (VC) and mean slope (MS) against plot-level mean values (cm) of DTM z-differences (N = 58). Significant sources of variation (*p* < 0.05) are presented in bold.

	Sum of Squares	Degrees of Freedom	Mean Squares	F-Statistic	*p*-Value
**Intercept**	**3472.547**	**1**	**3472.547**	**16.15292**	**0.000220**
**G**	**1861.025**	**3**	**620.342**	**2.88558**	**0.045942**
Lh	471.963	3	157.321	0.73179	0.538474
**VC**	**5471.562**	**3**	**1823.854**	**8.48385**	**0.000141**
MS	93.032	3	31.011	0.14425	0.932832
Error	9674.079	45	214.980		

**Table 6:** Four-way ANOVA of basal area (G), Lorey’s mean height (Lh), vegetation coverage (VC) and mean slope (MS) against plot-level standard deviation values (cm) of DTM z-differences (N = 58). Significant sources of variation (*p* < 0.05) are presented in bold.

	Sum of Squares	Degrees of Freedom	Mean Squares	F-Statistic	*p*-Value
**Intercept**	**18506.07**	**1**	**18506.07**	**38.44031**	**0.000000**
**G**	**4666.75**	**3**	**1555.58**	**3.23121**	**0.031015**
Lh	918.13	3	306.04	0.63570	0.595911
**VC**	**20574.84**	**3**	**6858.28**	**14.24583**	**0.000001**
MS	730.66	3	243.55	0.50590	0.680180
Error	21664.07	45	481.42		

**Table 7:** Four-way ANOVA of basal area (G), Lorey’s mean height (Lh), vegetation coverage (VC) and mean slope (MS) against plot-level L90 values (cm) of DTM z-differences (N = 58). Significant sources of variation (*p* < 0.05) are presented in bold.

	Sum of Squares	Degrees of Freedom	Mean Squares	F-Statistic	*p*-Value
**Intercept**	**64467.98**	**1**	**64467.98**	**34.36943**	**0.000000**
**G**	**18893.67**	**3**	**6297.89**	**3.35756**	**0.026893**
Lh	3710.72	3	1236.91	0.65942	0.581325
**VC**	**75276.48**	**3**	**25092.16**	**13.37723**	**0.000002**
MS	2211.79	3	737.26	0.39305	0.758583
Error	84408.13	45	1875.74		

**Table 8:** Unequal Tukey’s test HSD means separation for basal area (G), Lorey’s mean height (Lh), vegetation coverage (VC) and mean slope (MS) according to some plot-level DTM accuracy statistics. For a given row, different letters between data in different columns indicate significant differences (*p* < 0.05). All figures are expressed in cm.

Source of Variation	Measured Statistic	Average Class 1	Average Class 2	Average Class 3	Mean Class 4
**G**	Plot-level mean	−0.13a	−1.63a	−1.62a	−18.08a
	Plot-level SD	7.66a	6.61a	11.60a	37.28a
	Plot-level L90	13.60a	11.39a	19.32a	71.85a
**Lh**	Plot-level mean	1.22a	−1.28a	−8.81a	−2.37a
	Plot-level SD	8.18a	6.64a	22.95a	9.30a
	Plot-level L90	14.49a	11.59a	42.72a	15.50a
**VC**	Plot-level mean	**−0.51a**	**−2.58a**	**−2.65a**	**−45.15b**
	Plot-level SD	**6.73a**	**7.83a**	**17.40a**	**94.45b**
	Plot-level L90	**11.77a**	**13.60a**	**27.56a**	**179.65b**
**MS**	Plot-level mean	−0.85a	−5.00a	−2.37a	−3.06a
	Plot-level SD	6.17a	17.66a	7.21a	12.87a
	Plot-level L90	10.68a	31.81a	12.78a	24.38a

G classes: {[0–5] (5–10] (10–15] and >15 m^2^/ha}. Lh classes: {[0–8] (8–13] (13–20] and >20 m}. VC classes: {[0–20] (20–40] (40–60] and >60%}. MS classes: {[0–12] (12–24] (24–40] and >40%}.

## References

[1] Dong J., Kaufmann R.K., Myneni R.B., Tucker C.J., Kauppi P.E., Liski J., Buermann W., Alexeyev V., Hughes M.K. (2003). Remote sensing estimates of boreal and temperate forest woody biomass: Carbon pools, sources, and sinks. Remote Sens. Environ..

[2] Beer C., Reichstein M., Tomelleri E., Ciais P., Jung M., Carvalhais N., Rödenbeck C., Arain M.A., Baldocchi D., Bonan G.B. (2010). Terrestrial Gross Carbon Dioxide Uptake: Global Distribution and Covariation with Climate. Science.

[3] Agrawal A., Nepstad D., Chhatre A. (2011). Reducing Emissions from Deforestation and Forest Degradation. Annu. Rev. Environ. Resour..

[4] Dixon R.K., Solomon A.M., Brown S., Houghton R.A., Trexier M.C., Wisniewski J. (1994). Carbon pools and flux of global forest ecosystems. Science.

[5] Houghton R.A., Nassikas A.A. (2018). Negative emissions from stopping deforestation and forest degradation, globally. Glob. Chang. Biol..

[6] Herold M., Johns T. (2007). Linking requirements with capabilities for deforestation monitoring in the context of the UNFCCC-REDD process. Environ. Res. Lett..

[7] Kollert W., Cherubini L. (2012). Teak Resources and Market Assessment 2010.

[8] Cañadas Á., Andrade-Candell J., Domínguez J.M., Molina C., Schnabel O., Vargas-Hernández J.J., Wehenkel C. (2018). Growth and Yield Models for Teak Planted as Living Fences in Coastal Ecuador. Forests.

[9] White J.C., Coops N.C., Wulder M.A., Vastaranta M., Hilker T., Tompalski P. (2016). Remote Sensing Technologies for Enhancing Forest Inventories: A Review. Can. J. Remote Sens..

[10] Jucker T., Caspersen J., Chave J., Antin C., Barbier N., Bongers F., Dalponte M., van Ewijk K.Y., Forrester D.I., Haeni M. (2017). Allometric equations for integrating remote sensing imagery into forest monitoring programmes. Glob. Chang. Biol..

[11] Furukawa Y., Ponce J. (2010). Accurate, Dense, and Robust Multiview Stereopsis. IEEE Trans. Pattern Anal. Mach. Intell..

[12] Micheletti N., Chandler J.H., Lane S.N., Cook S.J., Clarke L.E., Nield J.M. (2015). Structure from motion (SFM) photogrammetry. Geomorphological Techniques (Online Edition).

[13] Smith M.W., Carrivick J.L., Quincey D.J. (2016). Structure from motion photogrammetry in physical geography. Prog. Phys. Geogr. Earth Environ..

[14] Moudrý V., Gdulová K., Fogl M., Klápště P., Urban R., Komárek J., Moudrá L., Štroner M., Barták V., Solský M. (2019). Comparison of leaf-off and leaf-on combined UAV imagery and airborne LiDAR for assessment of a post-mining site terrain and vegetation structure: Prospects for monitoring hazards and restoration success. Appl. Geogr..

[15] Remondino F., Spera M.G., Nocerino E., Menna F., Nex F. (2014). State of the art in high density image matching. Photogramm. Rec..

[16] Dandois J.P., Ellis E.C., Dandois J.P., Ellis E.C. (2010). Remote Sensing of Vegetation Structure Using Computer Vision. Remote Sens..

[17] White J., Wulder M., Vastaranta M., Coops N., Pitt D., Woods M. (2013). The Utility of Image-Based Point Clouds for Forest Inventory: A Comparison with Airborne Laser Scanning. Forests.

[18] Dandois J.P., Ellis E.C. (2013). High spatial resolution three-dimensional mapping of vegetation spectral dynamics using computer vision. Remote Sens. Environ..

[19] Goodbody T.R.H., Coops N.C., Marshall P.L., Tompalski P., Crawford P. (2017). Unmanned aerial systems for precision forest inventory purposes: A review and case study. For. Chron..

[20] Lisein J., Pierrot-Deseilligny M., Bonnet S., Lejeune P., Lisein J., Pierrot-Deseilligny M., Bonnet S., Lejeune P. (2013). A Photogrammetric Workflow for the Creation of a Forest Canopy Height Model from Small Unmanned Aerial System Imagery. Forests.

[21] Tang L., Shao G. (2015). Drone remote sensing for forestry research and practices. J. For. Res..

[22] Jensen J., Mathews A. (2016). Assessment of Image-Based Point Cloud Products to Generate a Bare Earth Surface and Estimate Canopy Heights in a Woodland Ecosystem. Remote Sens..

[23] Wallace L., Lucieer A., Malenovskỳ Z., Turner D., Vopěnka P. (2016). Assessment of forest structure using two UAV techniques: A comparison of airborne laser scanning and structure from motion (SfM) point clouds. Forests.

[24] Goodbody T.R.H., Coops N.C., Hermosilla T., Tompalski P., Crawford P. (2018). Assessing the status of forest regeneration using digital aerial photogrammetry and unmanned aerial systems. Int. J. Remote Sens..

[25] Mlambo R., Woodhouse I.H., Gerard F., Anderson K. (2017). Structure from motion (SfM) photogrammetry with drone data: A low cost method for monitoring greenhouse gas emissions from forests in developing countries. Forests.

[26] Kuželka K., Surový P. (2018). Mapping Forest Structure Using UAS inside Flight Capabilities. Sensors.

[27] Aguilar F.J., Nemmaoui A., Aguilar M.A., Peñalver A. (2019). Fusion of Terrestrial Laser Scanning and RPAS Image-based Point Clouds in Mediterranean Forest Inventories. DYNA.

[28] Newnham G.J., Armston J.D., Calders K., Disney M.I., Lovell J.L., Schaaf C.B., Strahler A.H., Mark Danson F. (2015). Terrestrial laser scanning for plot-scale forest measurement. Curr. For. Rep..

[29] Trochta J., Krůček M., Vrška T., Král K. (2017). 3D Forest: An application for descriptions of three-dimensional forest structures using terrestrial LiDAR. PLoS ONE.

[30] Moudrý V., Urban R., Štroner M., Komárek J., Brouček J., Prošek J. (2019). Comparison of a commercial and home-assembled fixed-wing UAV for terrain mapping of a post-mining site under leaf-off conditions. Int. J. Remote Sens..

[31] Flores Velasteguí T., Cabezas Guerrero F., Crespo Gutiérrez R. (2010). Plagas y enfermedades en plantaciones de Teca (*Tectona grandis* L.F.) en la zona de Balzar, provincia de Guayas. Cienc. y Tecnol..

[32] Holdridge L.R. (1982). Ecología Basada en Zonas de Vida.

[33] Rueda-Ayala V., Peña J., Höglind M., Bengochea-Guevara J., Andújar D., Rueda-Ayala V.P., Peña J.M., Höglind M., Bengochea-Guevara J.M., Andújar D. (2019). Comparing UAV-Based Technologies and RGB-D Reconstruction Methods for Plant Height and Biomass Monitoring on Grass Ley. Sensors.

[34] Panagiotidis D., Abdollahnejad A., Surový P., Chiteculo V. (2017). Determining tree height and crown diameter from high-resolution UAV imagery. Int. J. Remote Sens..

[35] Lucieer A., Turner D., King D.H., Robinson S.A. (2014). Using an Unmanned Aerial Vehicle (UAV) to capture micro-topography of Antarctic moss beds. Int. J. Appl. Earth Obs. Geoinf..

[36] Aguilar F.J., Mills J.P., Delgado J., Aguilar M.A., Negreiros J.G., Pérez J.L. (2010). Modelling vertical error in LiDAR-derived digital elevation models. ISPRS J. Photogramm. Remote Sens..

[37] Axelsson P. (2000). DEM generation from laser scanner data using adaptive TIN models. Int. Arch. Photogramm. Remote Sens..

[38] Felicísimo A.M. (1994). Parametric statistical method for error detection in digital elevation models. ISPRS J. Photogramm. Remote Sens..

[39] Aguilar F.J., Aguilar M.A., Blanco J.L., Nemmaoui A., Lorca A.M.G. (2016). Analysis and validation of grid DEM generation based on Gaussian markov random field. Int. Arch. Photogramm. Remote Sens. Spat. Inf. Sci. ISPRS Arch..

[40] Kershaw J.A., Ducey M.J., Beers T.W., Husch B. (2016). Forest Mensuration.

[41] Næsset E. (1997). Determination of mean tree height of forest stands using airborne laser scanner data. ISPRS J. Photogramm. Remote Sens..

[42] Muir J., Goodwin N.R., Armston J., Phinn S., Scarth P. (2017). An Accuracy Assessment of Derived Digital Elevation Models from Terrestrial Laser Scanning in a Sub-Tropical Forested Environment. Remote Sens..

[43] Daniel C., Tennant K., Maune D.F. (2001). DEM quality assessment. Digital Elevation Model Technologies and Applications: The DEM Users Manual.

[44] Aguilar M.A., Saldaña M.M., Aguilar F.J. (2014). Generation and Quality Assessment of Stereo-Extracted DSM From GeoEye-1 and WorldView-2 Imagery. IEEE Trans. Geosci. Remote Sens..

[45] Spjøtvoll E., Stoline M.R. (1973). An Extension of the T-Method of Multiple Comparison to Include the Cases with Unequal Sample Sizes. J. Am. Stat. Assoc..

[46] Baltsavias E.P. (1999). A comparison between photogrammetry and laser scanning. ISPRS J. Photogramm. Remote Sens..

[47] Chen Q., Gong P., Baldocchi D., Tian Y.Q. (2007). Estimating Basal Area and Stem Volume for Individual Trees from Lidar Data. Photogramm. Eng. Remote Sens..

[48] Tran-Ha M., Cordonnier T., Vallet P., Lombart T. (2011). Estimation du volume total aérien des peuplements forestiers à partir de la surface terrière et de la hauteur de Lorey. Rev. For. Française.

[49] Duursma R.A., Medlyn B.E. (2012). MAESPA: A model to study interactions between water limitation, environmental drivers and vegetation function at tree and stand levels, with an example application to [CO_2_] × drought interactions. Geosci. Model Dev..

[50] Keenan T., García R., Friend A.D., Zaehle S., Gracia C., Sabate S. (2009). Improved understanding of drought controls on seasonal variation in Mediterranean forest canopy CO_2_ and water fluxes through combined in situ measurements and ecosystem modelling. Biogeosciences.

[51] Kraus K., Pfeifer N. (1998). Determination of terrain models in wooded areas with airborne laser scanner data. ISPRS J. Photogramm. Remote Sens..

[52] Su J., Bork E. (2006). Influence of Vegetation, Slope, and Lidar Sampling Angle on DEM Accuracy. Photogramm. Eng. Remote Sens..

[53] Goodwin N.R., Coops N.C., Culvenor D.S. (2006). Assessment of forest structure with airborne LiDAR and the effects of platform altitude. Remote Sens. Environ..

[54] Tinkham W.T., Smith A.M.S., Hoffman C., Hudak A.T., Falkowski M.J., Swanson M.E., Gessler P.E. (2012). Investigating the influence of LiDAR ground surface errors on the utility of derived forest inventories. Can. J. For. Res..

[55] White J.C., Tompalski P., Coops N.C., Wulder M.A. (2018). Comparison of airborne laser scanning and digital stereo imagery for characterizing forest canopy gaps in coastal temperate rainforests. Remote Sens. Environ..

[56] Reutebuch S.E., McGaughey R.J., Andersen H.-E., Carson W.W. (2003). Accuracy of a high-resolution lidar terrain model under a conifer forest canopy. Can. J. Remote Sens..

[57] Hodgson M.E., Bresnahan P. (2004). Accuracy of Airborne Lidar-Derived Elevation. Photogramm. Eng. Remote Sens..

[58] Puttock A.K., Cunliffe A.M., Anderson K., Brazier R.E. (2015). Aerial photography collected with a multirotor drone reveals impact of Eurasian beaver reintroduction on ecosystem structure. J. Unmanned Veh. Syst..

[59] Meng X., Shang N., Zhang X., Li C., Zhao K., Qiu X., Weeks E., Meng X., Shang N., Zhang X. (2017). Photogrammetric UAV Mapping of Terrain under Dense Coastal Vegetation: An Object-Oriented Classification Ensemble Algorithm for Classification and Terrain Correction. Remote Sens..

[60] Ota T., Ogawa M., Shimizu K., Kajisa T., Mizoue N., Yoshida S., Takao G., Hirata Y., Furuya N., Sano T. (2015). Aboveground Biomass Estimation Using Structure from Motion Approach with Aerial Photographs in a Seasonal Tropical Forest. Forests.

[61] Yilmaz V., Konakoglu B., Serifoglu C., Gungor O., Gökalp E. (2018). Image classification-based ground filtering of point clouds extracted from UAV-based aerial photos. Geocarto Int..

[62] Granholm A.-H., Olsson H., Nilsson M., Allard A., Holmgren J. (2015). The potential of digital surface models based on aerial images for automated vegetation mapping. Int. J. Remote Sens..

[63] Fraser B., Congalton R. (2018). Issues in Unmanned Aerial Systems (UAS) Data Collection of Complex Forest Environments. Remote Sens..

[64] Véga C., Durrieu S., Morel J., Allouis T. (2012). A sequential iterative dual-filter for Lidar terrain modeling optimized for complex forested environments. Comput. Geosci..

[65] Smith S.L., Holland D.A., Longley P.A. (2005). Quantifying Interpolation Errors in Urban Airborne Laser Scanning Models. Geogr. Anal..

[66] Aguilar F.J., Agüera F., Aguilar M.A., Carvajal F. (2005). Effects of terrain morphology, sampling density, and interpolation methods on grid DEM accuracy. Photogramm. Eng. Remote Sens..

[67] Fisher P.F., Tate N.J. (2006). Causes and consequences of error in digital elevation models. Prog. Phys. Geogr. Earth Environ..

[68] Karel W., Kraus K., Höhle J., Potuckova M. (2006). Quality parameters of digital terrain models. The EuroSDR Test Checking and Improving of Digital Terrain Models.

[69] Aguilar F.J., Aguilar M.A., Agüera F., Sánchez J. (2006). The accuracy of grid digital elevation models linearly constructed from scattered sample data. Int. J. Geogr. Inf. Sci..

